# Mirror therapy for upper limb motor recovery after stroke: a meta-analysis

**DOI:** 10.3389/fneur.2026.1892502

**Published:** 2026-10-06

**Authors:** Yi Man, XiaoPei Liu, Xinyi Feng

**Affiliations:** 1 School of Rehabilitation Medicine, School of Nursing, Jiamusi University, Jiamusi, China; 2 The Third Affiliated Hospital of Jiamusi University, Jiamusi, China

**Keywords:** Fugl-Meyer assessment, Meta-analysis, mirror therapy, stroke, upper limb motor function

## Abstract

**Objective:**

To systematically evaluate the rehabilitation effect of mirror therapy (MT) on upper limb motor function in stroke patients, thereby providing evidence-based support for clinical rehabilitation decision-making.

**Methods:**

Randomized controlled trials (RCTs) investigating MT for upper limb dysfunction after stroke were retrieved from the PubMed, Web of Science, Cochrane Library, and EMBASE databases. The risk of bias in the included studies was assessed using the Cochrane Risk of Bias tool 2.0 (RoB 2.0). A meta-analysis was performed, and the grade of evidence was determined using the Grading of Recommendations Assessment, Development and Evaluation (GRADE) system.

**Results:**

A total of 21 RCTs were ultimately included. The meta-analysis showed that MT significantly improved the total score of the Fugl-Meyer Assessment Upper Extremity (FMA-UE) in stroke patients (Mean Difference [MD] = 5.45, 95% Confidence Interval [95% CI]: 3.50–7.41, *p* < 0.001). Hand function showed a statistically significant but clinically modest improvement (MD = 1.16, 95% CI: 0.03–2.29, *p* = 0.04); however, sensitivity analysis indicated that this statistical significance was not robust and depended on a small number of high-weight studies. A positive trend toward improvement was observed for shoulder-elbow-forearm function (MD = 1.79, 95% CI: −0.02 to 3.60, *p =* 0.05). However, no statistically significant improvement was found for wrist joint function (MD = 0.65, 95% CI: −0.17 to 1.47, *p =* 0.12). Heterogeneity was low across outcomes. No clear evidence of publication bias was detected; however, the number of studies was small for some outcomes, limiting the power of funnel plots and Egger’s test. The overall quality of evidence was moderate to low.

**Conclusion:**

MT may improve global upper limb motor function in stroke patients, with a more modest and less robust effect on hand function. The statistical significance of the hand-function finding depended on a small number of high-weight studies, and the evidence for hand function is of low quality; the overall evidence is low-to-moderate. Therefore, these findings, especially those for hand function, should be interpreted cautiously, and further high-quality studies are warranted.

**Systematic review registration:**

https://www.crd.york.ac.uk/prospero/display_record.php?ID=CRD420261395137, PROSPERO registration number: CRD420261395137.

## Introduction

Post-stroke upper extremity motor dysfunction remains a core challenge in rehabilitation medicine. Survivors exhibit varying degrees of residual upper limb impairment. Among these deficits, the loss of fine hand movements, shoulder-elbow coordination, and wrist joint stability severely restricts patients’ activities of daily living and quality of life (1). Although conventional rehabilitation therapies—such as physical therapy, occupational therapy, and constraint-induced movement therapy—have demonstrated efficacy, their effect on upper limb function improvement in patients with severe hemiplegia remains limited. Furthermore, the training process is often tedious and associated with poor adherence (2, 3). Consequently, exploring novel, more effective, feasible, and patient-acceptable rehabilitation approaches carries significant clinical importance.

Mirror therapy (MT) is a neurorehabilitation technique based on visual feedback. By creating a mirror illusion from the patient’s unaffected limb movements, MT activates the corresponding motor cortex of the affected hemisphere, thereby promoting neuroplastic repair (4). This method is simple to administer, low in cost, and not limited by the patient’s active movement ability. In recent years, MT has gained widespread attention in the field of post-stroke upper limb rehabilitation (5).

Previous systematic reviews and meta-analyses have investigated the efficacy of MT in improving upper extremity motor function in stroke patients. Saragih et al. confirmed that MT significantly improves overall upper limb motor function and hand function. Their subgroup analysis indicated that high-frequency, short-duration protocols yielded better outcomes; however, evidence regarding shoulder, elbow, forearm, and wrist joint function remained insufficient (6). Thieme et al. published a classic Cochrane systematic review that included large-sample studies and established the positive effects of MT in stroke rehabilitation with moderate-quality evidence, providing a core evidence base for clinical application (7). Nogueira et al. contributed complementary evidence from a neurophysiological perspective, noting that MT’s efficacy is primarily observed in the subacute phase and that MT can induce cortical reorganization in relevant brain regions (8). Boening et al. further refined intervention protocol differences, confirming that unilateral MT has clear efficacy whereas bilateral MT confers almost no additional benefit (9). Despite existing studies confirming the potential value of MT, heterogeneity persists across studies, and the strength of evidence for functional improvement in different upper limb segments varies significantly.

Therefore, this meta-analysis aims to systematically evaluate the effects of MT on the total score of the Fugl-Meyer Assessment Upper Extremity (FMA-UE) and its subscores for shoulder/elbow/forearm, wrist, and hand in stroke patients. Additionally, the certainty of the available evidence will be assessed to provide updated evidence-based support for clinical rehabilitation decision-making.

## Methods

### Literature screening and data extraction

#### Eligibility criteria

(1) Inclusion criteria

Study type: Only randomized controlled trials (RCTs) published in English were included.

Participants: Stroke patients diagnosed by computed tomography (CT) or magnetic resonance imaging (MRI) were included, with no restrictions on disease stage. Patients with severe cognitive impairment, aphasia preventing compliance with instructions, severe joint contractures or pain in the upper limb, or prior MT training were excluded.

Interventions: The experimental group received MT, which could be administered alone or in combination with conventional rehabilitation, occupational therapy, or physical therapy. No restrictions were placed on intervention frequency, duration per session, or total treatment period.

Comparators: The control group received conventional rehabilitation, sham MT, conventional occupational therapy, constraint-induced movement therapy, functional electrical stimulation, or other active interventions, provided that no form of MT was included.

Outcomes: At least one of the following outcomes was reported: upper limb motor function score, activities of daily living, grip strength, or motor activity log. This meta-analysis preferentially pooled data using FMA-UE–related outcomes.

(2) Exclusion criteria

Participants not clearly diagnosed with stroke or with mixed other neurological disorders (e.g., Parkinson’s disease, multiple sclerosis).

Intervention other than MT (e.g., pure action observation, virtual reality without mirror feedback).

No control group, or the control group also received MT.

No extractable outcome data reported, or data with obvious errors.

Duplicate publications or overlapping data (only the study with the largest sample size or most complete information was included).

Full text not available.

### Information sources

The following electronic databases were systematically searched: PubMed, Web of Science, Cochrane Library, and EMBASE. The search cut-off date for all databases was May 13, 2026. Additionally, reference lists of included studies and relevant systematic reviews/meta-analyses were manually searched to identify potentially missed studies. Clinical trial registries, gray literature, or professional society websites were not searched.

### Search strategy

Two researchers independently developed and executed the search strategy. Search terms combined controlled vocabulary and free text. Using PubMed as an example, the core search strategy was as follows: Intervention-related terms included “Mirror Therapy,” “mirror therapy,” “mirror visual feedback,” “mirror training,” or “mirror treatment.” Disease-related terms included “Stroke,” “stroke,” “cerebrovascular accident,” “hemiplegia,” “brain ischemia,” or “intracranial hemorrhage.” Outcome-related terms included “Upper Extremity,” “upper extremity,” “arm,” “hand,” “motor function,” “Fugl-Meyer,” or “FMA.” The detailed search strings and field settings for each database are presented in the Supplementary Table S1. To minimize the risk of missing relevant studies, the reference lists of included articles and related systematic reviews/meta-analyses were additionally hand-searched to identify potentially eligible studies not captured by the electronic database search. The protocol for this systematic review and meta-analysis was developed in accordance with the PRISMA-P guidelines (10) and registered with the International Prospective Register of Systematic Reviews (PROSPERO) under registration number [CRD420261395137]. The completed systematic review and meta-analysis was reported in accordance with the PRISMA 2020 statement.

### Study selection process

Search results from each database were imported into EndNote 21 reference management software, deduplicated automatically, and then manually verified. Two reviewers with training in evidence-based medicine independently performed a two-stage screening process. First stage: Titles and abstracts were screened against the eligibility criteria to exclude obviously irrelevant records. Second stage: Full texts of potentially eligible studies were retrieved and evaluated in detail for study design, population, intervention, comparator, and outcomes. If disagreements arose between the two reviewers, they were first resolved through discussion. If consensus could not be reached, a third senior researcher made the final decision. Reasons for full-text exclusion were recorded, and the entire screening process is illustrated in a PRISMA flow diagram.

### Data collection process

Two reviewers independently extracted information using a pre-designed and pilot-tested Excel data extraction form. Extracted items included: basic study information (first author, publication year, country), sample size, stroke type and duration, specific MT protocol (frequency, duration per session, total duration, and whether combined with other therapies), comparator details, and outcome measures (definition, time point of measurement, score value, and standard deviation). When data were incomplete or ambiguous, original authors were not contacted; instead, reasonable estimates were made based on reported information (e.g., extracting means and standard deviations from figures, taking the average of two reviewers’ estimates). If reliable values could not be obtained, the outcome was marked as “not reported” and excluded from the corresponding analysis. After cross-checking, disagreements were resolved through discussion or third-party arbitration.

### Data items

The following outcome variables were pre-specified for collection:

Primary outcomes: Total FMA-UE score (0–66) and available subscores (shoulder/elbow/forearm: 0–36; wrist: 0–10; hand: 0–14). To ensure consistency across studies, we prioritized data from the first post-intervention assessment (immediate post-treatment). If a study did not report immediate post-intervention scores, we extracted data from the longest available follow-up time point instead.

Other variables: Study characteristics (authors, year, country), stroke stage (acute: <1 month; subacute: 1–6 months; chronic: >6 months), total MT duration (minutes), comparator type (sham mirror/conventional rehabilitation/other), sample size, and baseline motor function scores. For missing baseline data (e.g., mean age standard deviation), “not reported” was noted descriptively without data imputation. When a study used multiple measurement tools for the same construct, only the data from the pre-specified tool were included.

### Bias and evidence quality assessment

#### Risk of bias in individual studies

The Cochrane Collaboration’s Risk of Bias 2.0 (RoB 2.0) tool for RCTs was used to evaluate each included RCT. This tool covers five domains: randomization process, deviations from intended interventions, missing outcome data, measurement of the outcome, and selective reporting of results. Two reviewers independently completed the assessment, assigning each domain a judgment of “low risk,” “some concerns,” or “high risk,” and then determined the overall risk of bias. Disagreements were resolved by discussion or by consulting a third researcher. No automated assessment tools were used.

#### Reporting bias

For the primary outcomes (total FMA-UE score and subscores), funnel plots were used for qualitative assessment to examine whether effect size estimates were symmetrically distributed around the pooled effect size. Furthermore, Egger’s linear regression test was performed for quantitative verification: the standard error of the effect size was used as the independent variable, and the effect size as the dependent variable. A significant regression intercept (*p* < 0.10) suggested possible small-study effects or publication bias. All analyses were performed based on the pooled results.

#### Certainty of evidence

The GRADE (Grading of Recommendations Assessment, Development and Evaluation) system was used to rate the certainty of evidence for each outcome. Two reviewers independently downgraded the evidence based on five domains: risk of bias, inconsistency, indirectness, imprecision, and publication bias. The final quality of evidence was classified as “high,” “moderate,” “low,” or “very low.” Disagreements were resolved through discussion or consultation with a methodologist.

### Effect measures and synthesis methods

#### Effect measures

All outcomes were continuous variables (FMA-UE scores), and the same rating scale was used across studies. Consequently, the effect measure was the mean difference (MD) with its 95% confidence interval (CI). If a study reported only medians, quartiles, and sample size, means and standard deviations were estimated using methods recommended in the literature. Effect measures from different scales were not combined.

#### Synthesis methods

First, basic characteristics, intervention protocols, and outcome data from each study were tabulated. Based on clinical and methodological homogeneity, a decision was made whether to perform a meta-analysis. For outcomes with ≥4 included studies and combinable data, meta-analysis was performed using RevMan 5.4 or Stata 17.0. Heterogeneity assessment: The *Q* test (*χ*^2^ distribution) and *I*^2^ statistic were used jointly. A *p* value < 0.10 or *I*^2^ > 50% was considered indicative of substantial heterogeneity. A fixed-effect model (inverse variance method) was pre-specified as the primary model to estimate a common intervention effect when the pre-specified eligibility criteria indicated that the included studies addressed the same clinical question and used comparable interventions and comparators. Random-effects sensitivity analyses were pre-planned to examine the robustness of the pooled estimates to potential clinical differences among studies. We also pre-specified that, if important statistical heterogeneity was present and could not be explained by subgroup analyses or other pre-specified sensitivity analyses, the random-effects results would also be used to interpret the findings. Subgroup analyses (pre-planned): exploratory subgroup analyses were planned according to stroke stage (acute/subacute/chronic), total MT duration (≤10 h vs. >10 h), and comparator type (sham mirror vs. conventional rehabilitation) to explore potential clinical effect modifiers, provided that sufficient data were available within each stratum. If data were insufficient within a stratum, the corresponding subgroup analysis was not performed. Snsitivity analysis: A leave-one-out approach (sequentially excluding one study at a time) was used to observe changes in the pooled effect estimate and assess the robustness of the results.

## Results

### Study selection results

The systematic search of four databases—PubMed (272 records), Web of Science (305 records), Cochrane Library (259 records), and Embase (82 records)—yielded a total of 918 records. After removing 152 duplicate records, 766 records remained for title and abstract screening. Among these, 715 records were excluded for failing to meet the inclusion criteria, leaving 51 full-text articles for assessment. During the full-text screening phase, 30 articles were excluded for not meeting the criteria for study type, participants, intervention, or outcome measures. Additionally, articles lacking complete outcome data, with duplicate publications, or with language constraints were excluded. Consequently, 21 studies were finally included (Supplementary Figure S1).

### Characteristics of included studies

The 21 included RCTs enrolled stroke patients across acute, subacute, and chronic stages. The MT intervention protocols and comparator interventions exhibited considerable diversity (Table 1). Geographically, the studies were predominantly conducted in Asia and Europe, providing good regional representation. Sample sizes varied widely across studies, with most ranging from 20 to 60 participants. All stroke stages were covered, with the subacute and chronic phases accounting for the highest proportions. Regarding interventions, the MT group most commonly received “MT combined with conventional rehabilitation,” with additional regimens including MT alone, task-oriented MT, and digital MT. Most control groups received conventional rehabilitation or sham MT, with some studies including multiple comparator arms. MT protocols varied; some studies reported individualized regimens with twice-daily interventions and explicitly stated total pure treatment durations. Overall, the baseline characteristics of the included studies showed substantial heterogeneity, covering MT application models across different clinical scenarios, thereby providing a foundation for comprehensively evaluating its rehabilitation effects. Nevertheless, some studies had small sample sizes and considerable variation in intervention protocols, which may have influenced the meta-analysis results.

**Table 1:** Basic characteristics of included studies.

Included study	Country/region	Total sample size	Stroke stage	Mean time since stroke (months)	Intervention (MT group)	Intervention (control group)	MT protocol
Alsalem 2025 (11)	Saudi Arabia	38	Chronic	20.24	MT + CPT	CPT	Pure MT time: 15 min/session; Total intervention time: 45 min/session (MT 15 min + CPT 30 min); 3 sessions/week for 6 weeks
Amasyali 2016 (6)	Turkey	24	Subacute	5.25	MT + CPT	ES group: EMG-triggered electrical stimulation + CPT; CG group: CPT	30 min/session, 5 sessions/week for 3 weeks
Antoniotti 2019 (12)	Italy	40	Acute/Subacute	0.76	MT + conventional rehabilitation	Sham MT + conventional rehabilitation	30 min/session, 5 sessions/week for 30 days
Arya 2015 (13)	India	33	Chronic	12.5	TBMT	Conventional occupational therapy	45 min/session (pure MT), 5 sessions/week for 8 weeks
Chan 2018 (14)	Hong Kong, China	41	Subacute	0.44	MT	Mirror absent, bilateral direct observation	30 min/session, 2 sessions/day, 5 days/week for 4 weeks (total pure MT time: 1200 min)
de Sire 2025 (15)	Italy	120	NA	NA	MT	CIMT group: modified constraint-induced movement therapy; Conventional group: conventional rehabilitation	45 min/session, 5 days/week for 40 days
Gurbuz 2016 (16)	Turkey	31	Subacute	1.48	MT	Sham MT	20 min/session, 5 days/week for 4 weeks + conventional rehabilitation (60–120 min/day)
Hsieh 2025 (17)	Taiwan, China	19	Chronic	22.7	Digital MT	DAOT group: digital action observation therapy; Conventional CR group: conventional occupational therapy	60 min/session, 15 sessions total over 3–4 weeks
Hsu 2022 (18)	Taiwan, China	54	Chronic	36.2	MT + COT	VRMT group: VRMT + COT; COT group: conventional occupational therapy + conventional task training	30 min/session, 2 sessions/week for 9 weeks
Kim 2023 (19)	South Korea	36	Subacute/Chronic	6.81	MT + conventional rehabilitation	CG: conventional rehabilitation only	30 min/session, 5 sessions/week for 4 weeks
Lee 2012 (20)	South Korea	26	Acute	3.55	MT + conventional rehabilitation	Standard rehabilitation	25 min/session, 2 sessions/day, 5 sessions/week for 4 weeks
Madhoun 2020 (21)	China	30	Subacute	NA	TBMT + conventional therapy	Conventional occupational therapy + conventional therapy	25 min/session for 25 sessions total
Michielsen 2011 (22)	Netherlands	40	Chronic	NA	TBMT + conventional therapy	Conventional rehabilitation	60 min/session, 5 days/week for 6 weeks
Mirela 2015 (23)	Romania	15	Subacute	NA	TBMT + conventional therapy	Conventional rehabilitation	30 min/session, 5 sessions/week for 6 weeks
Radajewska 2013 (24)	Poland	60	Subacute	NA	MT + conventional rehabilitation	Conventional rehabilitation	15 min/session, 2 sessions/day, 5 days/week for 3 weeks
Samuelkamaleshkumar 2014 (25)	India	20	Subacute	0.93	MT + conventional	Conventional rehabilitation	30 min/session, 2 sessions/day, 5 days/week for 3 weeks
Wen 2022 (26)	China	52	Acute/Subacute	1.03	MT + OT	OT	30 min/session, 6 sessions/week for 3 weeks
Wu 2013 (27)	Taiwan, China	33	Chronic	20.6	MT + task-oriented training	Task-oriented training	60 min/session, 5 days/week for 4 weeks
Yavuzer 2008 (28)	Turkey	40	Subacute	5.45	MT + conventional rehabilitation	Sham MT + conventional rehabilitation	30 min/session, 5 days/week for 4 weeks
Zhang 2021 (29)	China	60	Acute/Subacute	1	MT + OT	OT	60 min/session, 5 days/week for 4 weeks
Zhang 2024 (30)	China	60	Subacute	0.78	MT + conventional rehabilitation	CCFES group: contralaterally controlled functional electrical stimulation; MT + CCFES group: MT combined with CCFES; Control group: conventional rehabilitation only	20 min/session, 5 days/week for 3 weeks

MT, mirror therapy; CPT, conventional physical therapy; TBMT, task-based mirror therapy; CIMT, constraint-induced movement therapy; OT, occupational therapy; CCFES, contralaterally controlled functional electrical stimulation; NA, not available.

Baseline demographic characteristics, disease characteristics, and functional status were generally well balanced between groups (Supplementary Table S2). Participant ages ranged from 48 to 71 years, with a higher proportion of males than females, consistent with the epidemiology of stroke. Ischemic stroke was the predominant type. The distribution of the affected side (left vs. right) was balanced. The disease course covered all stages. No significant between-group differences were observed at baseline in cognitive function, muscle tone, or upper limb motor function, thereby minimizing the main confounding effect of baseline differences on intervention effect estimation. Nevertheless, some studies had missing key baseline data or inconsistent reporting formats for certain indicators. Despite these limitations, the overall evidence provided important support for the reliability of the meta-analysis results.

### Results of risk of bias in individual studies

The Cochrane Risk of Bias Tool 2.0 (ROB 2.0) was used to assess the risk of bias in the included randomized controlled trials (Supplementary Figure S2). The assessment yielded the following results: In the domain of randomization process, 15 studies were rated as low risk, and 6 studies raised some concerns. Regarding deviations from intended interventions, 14 studies were rated as low risk, and 7 studies raised some concerns. For missing outcome data, 17 studies were rated as low risk, 2 studies raised some concerns, and 2 studies were rated as high risk. In the outcome measurement domain, 18 studies were rated as low risk, and 3 studies raised some concerns. For the selection of the reported result, all 21 studies were rated as low risk. Overall, 10 studies were rated as low risk of bias, 9 raised some concerns, and 2 were rated as high risk. Consequently, the methodological quality of the included studies was moderately high, and the risk of bias was within an acceptable range, thereby providing a foundational guarantee for the reliability of this meta-analysis.

### Results of individual studies and synthesis results

#### FMA-UE score

For all forest plots, the experimental group (MT) is displayed on the left and the control group on the right, and the mean difference (MD) is calculated as MT minus control. Therefore, a positive MD indicates a beneficial effect of MT. The heterogeneity test showed no statistical heterogeneity among the studies (Chi^2^ = 12.97, df = 13, *p =* 0.45, *I*^2^ = 0%). Therefore, a fixed-effects model using the inverse variance method was employed for the pooled effect size analysis (Figure 1). The pooled results indicated that, compared with the control group, mirror therapy (MT) significantly improved the FMA-UE score in stroke patients, with a mean difference (MD) of 5.45 (95% CI [3.50, 7.41]). The overall effect test was highly statistically significant (*Z* = 5.47, *p* < 0.001).

**Figure 1:**
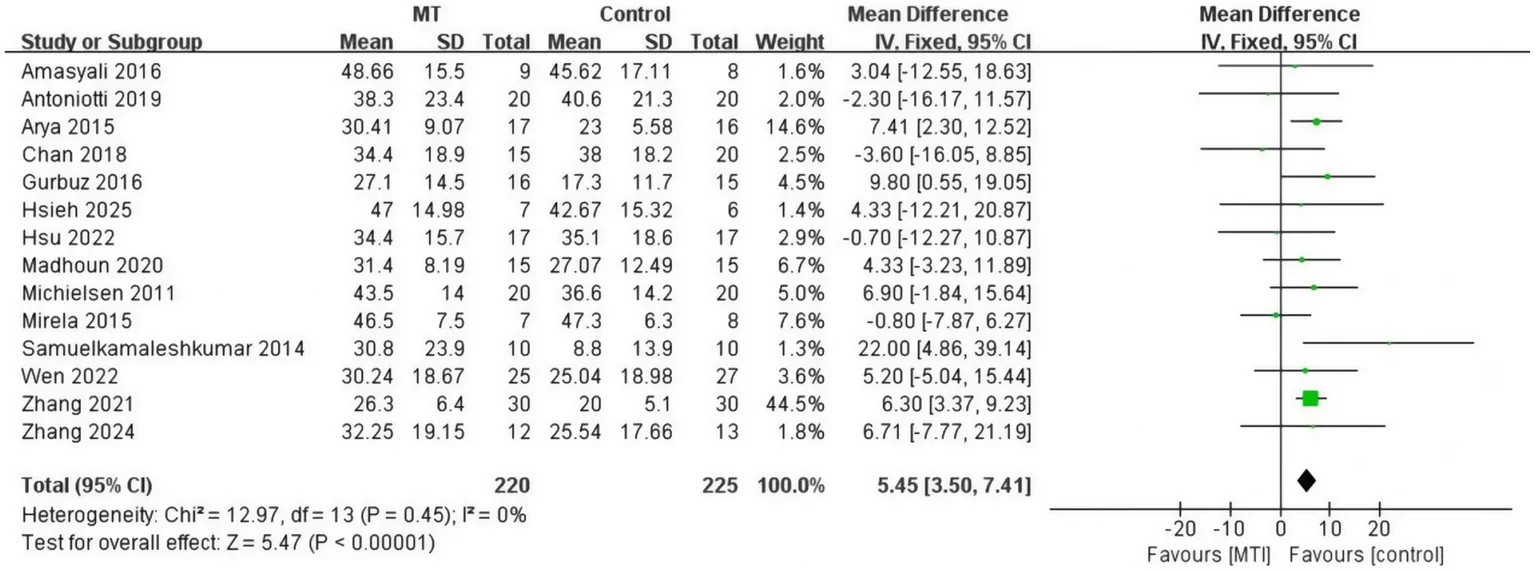
Forest plot comparing FMA-UE scores.

Analysis of individual study results revealed that four studies individually showed significantly higher FMA-UE scores in the MT group than in the control group. The remaining 10 studies did not show statistically significant differences individually; however, the direction of the effect in most studies was consistent with the pooled result. The heterogeneity among studies was extremely low (*I*^2^ = 0%), suggesting good consistency across study results.

Sensitivity analysis showed that after sequentially omitting each study, the recalculated pooled MD ranged from 4.77 to 5.97, and the 95% CI never crossed the line of no effect (0). The overall effect test remained highly statistically significant in all recalculations (*p* < 0.001). Furthermore, the heterogeneity index *I*^2^ remained between 0 and 10% across all re-analyses. Random-effects sensitivity analyses were also performed for outcome; the random-effects estimates were consistent with the fixed-effect estimates, and the conclusions remained unchanged.

#### Subgroup analysis by stroke stage

Although the overall heterogeneity was low, we performed the preplanned subgroup analyses according to the available stratification data to explore potential clinical effect modifiers. For FMA-UE total score, stratification was feasible based on the reported stroke phases. The results showed that MT significantly improved FMA-UE scores across the different strata, with no statistically significant interaction between subgroups (*P* for interaction = 1.00), suggesting that stroke stage did not substantially modify the overall treatment effect. For the other outcome measures (shoulder/elbow/forearm, wrist, and hand subscores), subgroup analyses could not be conducted due to an insufficient number of studies within each stratum. These exploratory findings should be interpreted with caution, as the number of studies in certain strata was limited.

#### FMA-UE shoulder/elbow/forearm subscale

The heterogeneity test showed no statistical heterogeneity among the studies (Chi^2^ = 3.09, df = 5, *p =* 0.69, *I*^2^ = 0%). Consequently, a fixed-effects model using the inverse variance method was used for the pooled effect size analysis (Figure 2). The pooled results showed a trend toward improvement in shoulder/elbow/forearm motor function with MT compared to the control group, with a pooled MD of 1.79 (95% CI [−0.02, 3.60]). The overall effect test approached statistical significance (*Z* = 1.93, *p =* 0.05).

**Figure 2:**
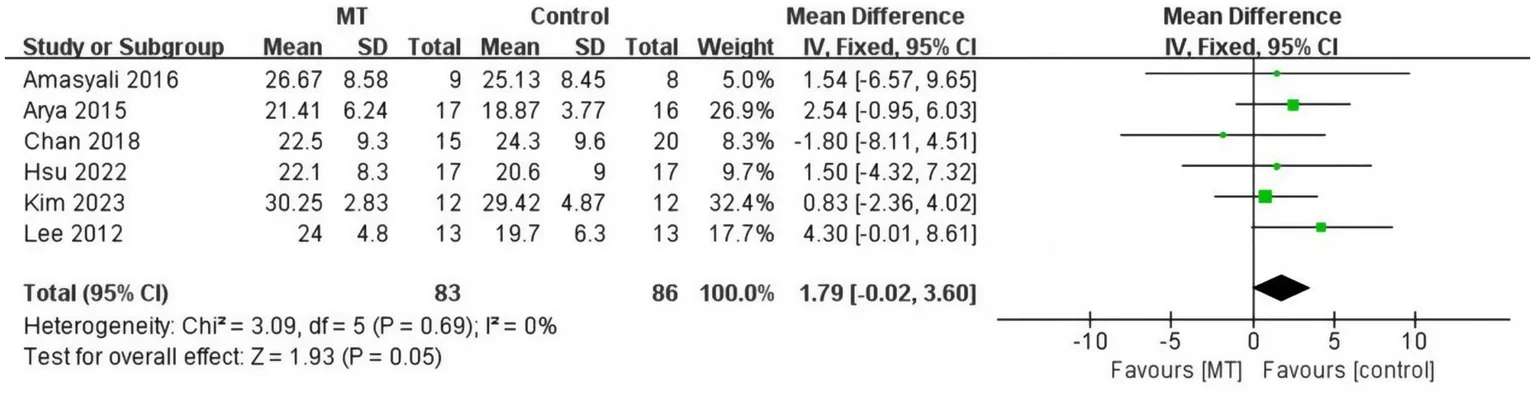
Forest plot comparing FMA-UE shoulder/elbow/forearm subscale scores.

Analysis of individual study results indicated that none of the six studies individually showed a statistically significant difference; nevertheless, the direction of the effect in most studies aligned with the pooled result.

Sensitivity analysis demonstrated that after sequentially omitting each study, the pooled MD ranged from 1.25 to 2.25. The heterogeneity index *I*^2^ remained at 0% across all analyses, consistent with the original finding. The direction of the pooled effect consistently favored the MT group without reversal. However, statistical significance showed some fluctuations: after omitting Chan et al., the result became significant (*p =* 0.03); after omitting Arya et al. or Lee et al., the result lost statistical significance (*p* > 0.05); after omitting other studies, the *p* values ranged from 0.05 to 0.06 (13, 14, 20). Random-effects sensitivity analyses were also performed for outcome; the random-effects estimates were consistent with the fixed-effect estimates, and the conclusions remained unchanged.

#### FMA-UE wrist subscale

The heterogeneity test showed no statistical heterogeneity among the studies (Chi^2^ = 2.88, df = 5, *p =* 0.72, *I*^2^ = 0%). Therefore, a fixed-effects model using the inverse variance method was used for the pooled effect size analysis. The pooled results showed a trend toward improvement in wrist joint motor function with MT compared to the control group, but this did not reach statistical significance. The pooled MD was 0.65 (95% CI [−0.17, 1.47]), and the overall effect test was not statistically significant (*Z* = 1.54, *p =* 0.12).

Analysis of individual study results indicated that none of the six studies individually showed a statistically significant difference. The point estimates of effect size favored the MT group in four studies and favored the control group in two studies; however, the 95% CIs of all studies included 0, indicating no statistical significance.

Sensitivity analysis showed that after sequentially omitting each study, the pooled MD ranged from 0.46 to 0.79. The heterogeneity index *I*^2^ remained at 0% across all analyses, consistent with the original finding. Furthermore, the direction of the pooled effect consistently favored the MT group without reversal. Regarding statistical significance, only after omitting Chan et al. did the result approach statistical significance (*p =* 0.07); after omitting other studies, the *p* values ranged from 0.12 to 0.35, showing no significant difference from the original result (*p =* 0.12) (14) (Figure 3). Random-effects sensitivity analyses were also performed for outcome; the random-effects estimates were consistent with the fixed-effect estimates, and the conclusions remained unchanged.

**Figure 3:**
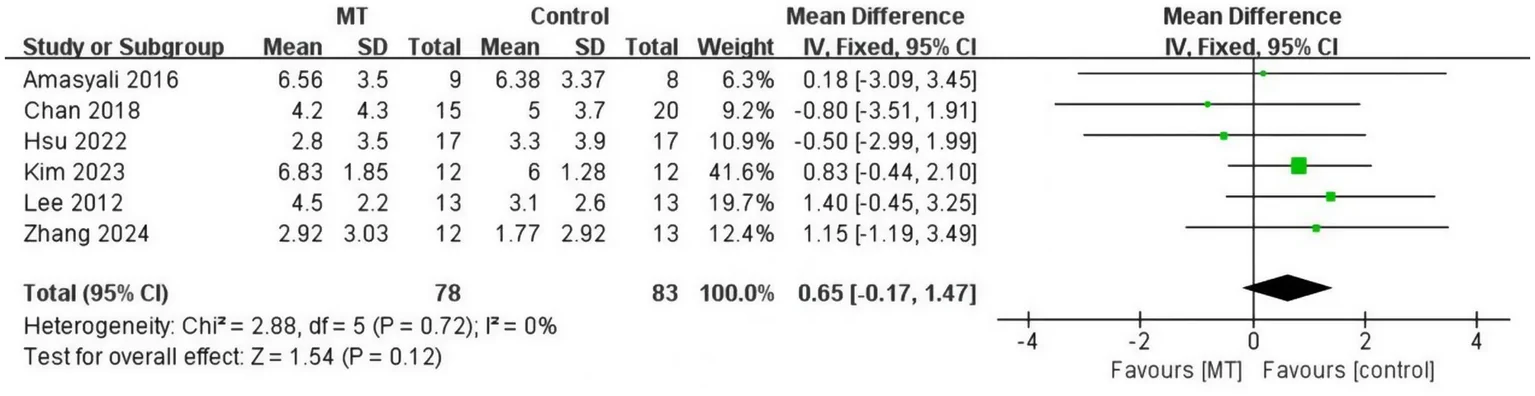
Forest plot comparing FMA-UE wrist subscale scores.

#### FMA-UE hand subscale

The heterogeneity test showed no statistical heterogeneity among the studies (Chi^2^ = 3.88, df = 4, *p =* 0.42, *I*^2^ = 0%). Consequently, a fixed-effects model using the inverse variance method was used for the pooled effect size analysis. The pooled results indicated that, compared with the control group, MT significantly improved the FMA-hand score in stroke patients, with a pooled MD of 1.16 (95% CI [0.03, 2.29]). The overall effect test was statistically significant (*Z* = 2.02, *p =* 0.04).

Analysis of individual study results showed that the direction of the effect in most studies was consistent with the pooled result. Specifically, two studies (Kim et al., Lee et al., 2012) had relatively large point estimates of effect size that approached statistical significance (19, 20). Two studies (Chan et al. and Hsu et al.) had point estimates favoring the control group, but their 95% CIs included 0, indicating no statistical significance (14, 18).

Sensitivity analysis demonstrated that after sequentially omitting each study, the pooled MD ranged from 0.86 to 1.41. The direction of the pooled effect consistently favored the MT group across all analyses without reversal. However, statistical significance showed notable fluctuations. After omitting Chan et al. or Hsu et al., the statistical significance of the result further strengthened (*p* < 0.05) (14, 18). After omitting Amasyali et al., the *p* value decreased to the critical value of 0.05 (6). By contrast, after omitting the highest-weight studies (Kim et al. or Lee et al.), the 95% CI crossed the line of no effect (0), and the original statistical significance disappeared (19, 20). Notably, in a few sensitivity analyses, the *I*^2^ slightly increased (up to 23%) compared to the original *I*^2^ of 0%, which can occur when a study that previously contributed to stabilizing between-study variance is removed. Importantly, all recalculated *I*^2^ values remained well below the 50% threshold, indicating consistently low heterogeneity and confirming the robustness of the main findings. Collectively, these results indicate that the overall direction of the effect in this meta-analysis of FMA-hand scores was stable, but the robustness of statistical significance was poor. The positive result primarily depended on the combined contribution of the two high-weight studies (Kim et al. and Lee et al.), with the study by Kim et al. having the most pronounced impact on the overall result (19, 20). Consequently, the evidence for an effect on hand function should be interpreted with caution, and no firm conclusion about clinical benefit can be drawn, and the improvement effect of MT on fine hand motor function in stroke patients requires further validation through more large-sample, multicenter, high-quality randomized controlled trials (Figure 4). Random-effects sensitivity analyses were also performed for outcome; the random-effects estimates were consistent with the fixed-effect estimates, and the conclusions remained unchanged.

**Figure 4:**
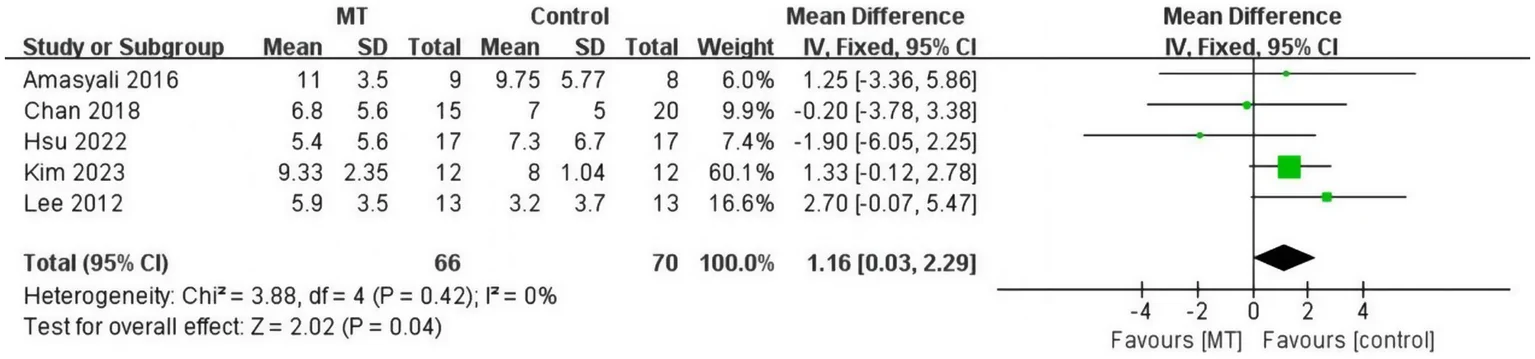
Forest plot comparing FMA-UE hand subscale scores.

#### Results of reporting bias

Funnel plots combined with Egger’s linear regression test were used to assess the risk of publication bias in this meta-analysis (Supplementary Figure S3).

The funnel plot for FMA-UE showed that the scatter points of the included studies were distributed roughly symmetrically around the pooled effect size, with most points concentrated in the middle and upper parts of the funnel plot. No obvious unilateral missing, truncation, or asymmetry was observed. Egger’s test results showed a regression intercept beta1 = −0.36 (SE = 0.504, *Z* = −0.71, *p =* 0.4763), failing to reject the null hypothesis of no small-study effects. These visual and quantitative assessments did not suggest clear evidence of publication bias; however, because the number of included studies was relatively small for some outcomes, the power of funnel plots and Egger’s test to detect small-study effects was limited, and publication bias cannot be entirely excluded.

The funnel plot for the FMA-UE shoulder/elbow/forearm subscale showed that the scatter points were distributed roughly symmetrically around the pooled effect size, with no obvious unilateral missing, truncation, or asymmetry. Egger’s test results showed a regression intercept beta1 = −0.52 (SE = 1.337, *Z* = −0.39, *p =* 0.6964), failing to reject the null hypothesis of no small-study effects. These visual and quantitative assessments were consistent, indicating no significant publication bias in this analysis.

The funnel plot for the FMA-wrist score showed that the scatter points were distributed roughly symmetrically around the pooled effect size, with no obvious unilateral missing, truncation, or asymmetry. Egger’s test results showed a regression intercept beta1 = −1.21 (SE = 1.300, *Z* = −0.93, *p =* 0.3515), failing to reject the null hypothesis of no small-study effects. These visual and quantitative assessments were consistent, indicating no significant publication bias in this analysis.

The funnel plot for the FMA-hand score showed that the scatter points were distributed roughly symmetrically around the pooled effect size, with no obvious unilateral missing, truncation, or asymmetry. Egger’s test results showed a regression intercept beta1 = −0.91 (SE = 1.041, *Z* = −0.87, *p =* 0.3835), failing to reject the null hypothesis of no small-study effects. These visual and quantitative assessments were consistent, indicating no significant publication bias in this analysis. Consequently, the results of this meta-analysis on the effect of MT on fine hand motor function in stroke patients are considered to have good reliability.

#### Results of certainty of evidence

The GRADE (Grading of Recommendations Assessment, Development and Evaluation) system was used to assess the certainty of evidence for the four outcome measures of MT for improving upper extremity motor function in stroke patients. The results showed that the evidence level for the FMA-UE total score was moderate. The evidence levels for the FMA-shoulder/elbow/forearm score, FMA-wrist score, and FMA-hand score were all low. The downgrading reasons were as follows: each outcome was downgraded by one level because important risk of bias was present among the studies contributing to that outcome. Additionally, the three subscale outcomes were downgraded by two levels due to serious imprecision (small sample sizes, 95% confidence intervals that crossed or approached the line of no effect). For the FMA-hand score, sensitivity analysis showed that its statistical significance depended on two high-weight studies, indicating poor result robustness. Overall, the quality of evidence in this meta-analysis was relatively low. Although the evidence for the improvement effect of MT on overall upper extremity motor function in stroke patients was relatively sufficient, the improvement effects on individual upper extremity sub-functions require further validation through more large-sample, high-quality randomized controlled trials (Table 2).

**Table 2:** Results of certainty of evidence.

Outcome measure	Risk of bias	Inconsistency	Indirectness	Imprecision	Publication bias	Certainty of evidence	Remarks
FMA-UE total score	Downgraded by 1 level	No downgrade	No downgrade	No downgrade	No downgrade	Moderate	10 studies low risk, 9 some concerns, 2 high risk; important bias present. Heterogeneity *I*^2^ = 0%, good consistency. 95% CI did not cross null line; adequate sample size; no significant publication bias
FMA-shoulder/elbow/forearm score	Downgraded by 1 level	No downgrade	No downgrade	Downgraded by 2 levels	No downgrade	Low	Important bias present. 95% CI crossed null line; effect size approached statistical significance threshold; small sample size; serious imprecision
FMA-wrist score	Downgraded by 1 level	No downgrade	No downgrade	Downgraded by 2 levels	No downgrade	Low	Important bias present. 95% CI crossed null line; not statistically significant; small sample size; serious imprecision
FMA-hand score	Downgraded by 1 level	No downgrade	No downgrade	Downgraded by 2 levels	No downgrade	Low	Important bias present. 95% CI approached null line; sensitivity analysis showed significance disappeared after omitting high-weight studies; small sample size; poor result robustness

FMA-UE, Fugl-Meyer Assessment for Upper Extremity; CI, confidence interval; GRADE, Grading of Recommendations Assessment, Development and Evaluation.

## Discussion

### Comprehensive interpretation of results

This meta-analysis systematically evaluated the effects of mirror therapy (MT) on upper extremity motor function recovery in patients with stroke. The results showed that, compared with control interventions, MT significantly improved overall upper extremity motor function. Specifically, improvements in fine hand motor function reached statistical significance in the main analysis, but this finding was not robust in sensitivity analysis and depended on a small number of high-weight studies; improvements in shoulder-elbow-forearm function approached the statistical threshold, and wrist joint function did not achieve statistical significance. Statistical heterogeneity was low for all outcome measures, and no clear evidence of publication bias was detected; however, the limited number of studies for some outcomes reduced the power to detect small-study effects, so publication bias cannot be excluded. The overall direction of the results was stable; however, the certainty of evidence for individual upper extremity subcomponents was relatively low.

From a neurophysiological perspective, the differential effects of MT on upper extremity subcomponents have a clear biological basis. The mirror neuron system is primarily distributed in the primary motor cortex, premotor cortex, and parietal lobe. The cortical representational area for hand movements is substantially larger than that for shoulder-elbow and wrist movements. Consequently, mirror visual feedback induces more pronounced activation of the hand motor cortex. These findings align with previous neurophysiological studies, which have demonstrated that MT can induce significant cortical reorganization in hand-related brain regions (31, 32). Furthermore, most MT protocols in clinical practice focus on fine hand movements such as grasping and pinching, whereas specific training for shoulder-elbow flexion/extension and wrist rotation is relatively insufficient. This disparity may also explain why hand function improved more significantly while wrist function did not show notable improvement (6).

The finding that shoulder-elbow-forearm function approached the statistical threshold carries important clinical implications. Sensitivity analyses indicated that some control measures themselves might have certain therapeutic effects through motor observation, thereby attenuating the between-group differences attributed to MT. Moreover, the included studies had a relatively high proportion of patients in the chronic phase. In these patients, shoulder and elbow joints often exhibit varying degrees of muscle atrophy and joint contracture. Consequently, the effects of MT in chronic patients were weaker than those in subacute patients, which may have lowered the overall effect size.

Of note, heterogeneity across studies was extremely low for all outcomes, significantly lower than reported in most previous meta-analyses. This can be largely attributed to the strict uniformity of outcome assessment tools applied in this study, which avoided methodological heterogeneity arising from different scales. Additionally, the core elements of the intervention protocols were highly consistent across included studies, with most studies combining MT with conventional rehabilitation. Thus, these results suggest that the therapeutic efficacy of MT is highly reproducible in standardized clinical application scenarios, providing more robust evidence for its widespread clinical adoption.

### Clinical significance, effect magnitude, and treatment parameter optimization

When interpreting the pooled FMA-UE improvement of 5.45 points, it is important to consider the minimal clinically important difference (MCID), which varies across stroke stages. Published estimates suggest MCID values of approximately 5.25–7.25 points for subacute stroke and 4.25–6.25 points for chronic stroke (33, 34). Our pooled estimate falls within the lower range of these thresholds, indicating that the observed improvement may be clinically meaningful for some patients but not consistently across all subgroups. For the hand subscale, the pooled MD of 1.16 points is below the MCID thresholds for hand-specific measures, and given the low certainty of evidence, this finding should be interpreted as exploratory rather than definitive. Future studies with larger samples are needed to confirm whether MT yields clinically meaningful improvements in hand function.

From a practical standpoint, based on the intervention protocols summarized from the included studies, the following recommendations for MT application in post-stroke upper limb rehabilitation can be derived. The majority of effective protocols employed a total pure MT duration of approximately 10–20 h, delivered as 20- to 60-min sessions (most commonly 30 min per session), with a frequency of 5 sessions per week or twice daily over a total treatment period of 3 to 6 weeks. These parameters were associated with significant improvements in overall upper limb function and hand function. However, it should be noted that the optimal dose–response relationship remains to be established, and treatment parameters should be individualized according to patient tolerance, stroke stage, and rehabilitation goals. For patients with severe impairment or fatigue, a lower starting dose with gradual titration is advisable. Clinicians are also encouraged to combine MT with conventional rehabilitation rather than using it as a standalone therapy, as this combination was the most frequently applied and effective approach in the included trials.

## Limitations

First, our search strategy was restricted to English-language publications and four major databases (PubMed, Web of Science, Cochrane Library, and EMBASE), without including other potential sources such as CINAHL, PEDro. This pragmatic constraint may have introduced language and retrieval bias, potentially overlooking relevant studies, particularly from East Asian regions or allied health fields. Nevertheless, the 21 included RCTs already covered diverse geographic settings, and the consistently low heterogeneity and robust sensitivity analyses suggest that these omissions are unlikely to have materially altered our core findings. Second, the methodological quality of included studies varied. Some studies had deficiencies in randomization, allocation concealment, and blinding. Blinding is particularly difficult to implement in MT, which may introduce measurement bias. Third, most studies were small-sample, single-center designs with insufficient statistical power. Consequently, the certainty of evidence for upper extremity subcomponent outcomes was low, and the robustness of results was suboptimal. Fourth, although we performed subgroup analyses, the intervention protocols (duration, frequency, training content, and co-interventions) were not fully standardized, precluding definitive determination of optimal treatment parameters. Fifth, the exclusion of patients with severe cognitive impairment or joint contractures limits the applicability of our conclusions to these complex clinical subgroups. Furthermore, we focused exclusively on FMA-UE and did not assess other important outcomes such as activities of daily living or long-term prognosis. Future trials should include underrepresented populations and patient-centered outcomes. Sixth, we did not contact authors to obtain missing or figure-only data; instead, unreported means and standard deviations were estimated using validated computational methods. While this may have marginally affected the precision of study-specific weights and confidence intervals, the low heterogeneity and robust sensitivity analyses suggest no material impact on the pooled estimates.

### Implications for practice, policy, and future research

In clinical practice, MT can be used as a routine adjunctive intervention for upper extremity rehabilitation after stroke. Priority should be given to improving overall upper extremity function and fine hand motor function. Meanwhile, targeted supplementary training for shoulder-elbow and wrist joints should be added to enhance overall rehabilitation outcomes. At the policy level, given its low cost and ease of administration, MT should be integrated into standardized stroke rehabilitation protocols, with efforts to promote its application in primary care rehabilitation facilities. Future research should include large-sample, multicenter randomized controlled trials, incorporate non-English studies and special populations such as severely affected or chronic patients, standardize intervention protocols, and conduct long-term follow-up to further improve evidence quality.

## Conclusion

Mirror therapy improves overall upper-extremity motor function in stroke patients and shows a promising trend for shoulder-elbow-forearm recovery, whereas its effect on wrist function remains unsubstantiated. The improvement in hand function reached statistical significance but was clinically modest and based on low-certainty evidence. Given these moderate-to-low certainty findings, we recommend MT as an adjunctive enhancement to, rather than a replacement for, conventional rehabilitation. Future high-quality, large-sample trials with standardized protocols and broader patient populations are warranted to validate the therapeutic effects and optimize intervention protocols.

## Data Availability

The datasets presented in this study can be found in online repositories. The names of the repository/repositories and accession number(s) can be found in the article/Supplementary material.
