# Supplementary material for: Mirror therapy for upper limb motor recovery after stroke: a meta-analysis
Source: Front Neurol. 2026 Oct 6;17:1892502. doi: 10.3389/fneur.2026.1892502 (PMC13680198; doi:10.3389/fneur.2026.1892502)
Supplement: Supplementary file 1 [file Supplementary_file_1.docx]

**Table S1.** Systematic literature review search terms and strategy.

**Search terms for PubMed**

#1 Mirror Therapy[Title/Abstract] OR mirror visual feedback[Title/Abstract] OR mirror training[Title/Abstract] OR mirror treatment[Title/Abstract]

#2 Stroke[Title/Abstract] OR cerebrovascular accident[Title/Abstract] OR hemiplegia[Title/Abstract] OR brain ischemia[Title/Abstract] OR intracranial hemorrhage[Title/Abstract]

#3 Upper Extremity[Title/Abstract] OR arm[Title/Abstract] OR hand[Title/Abstract] OR motor function[Title/Abstract] OR Fugl-Meyer[Title/Abstract] OR FMA[Title/Abstract]

#4 Randomized Controlled Trial[pt] OR RCT[Title/Abstract] OR randomized[Title/Abstract]

#5 #1 AND #2 AND #3 AND #4

**Search terms for Embase**

#1 'Mirror Therapy'/exp OR mirror visual feedback.mp. OR mirror training.mp. OR mirror treatment.mp.

#2 'Stroke'/exp OR cerebrovascular accident.mp. OR hemiplegia.mp. OR brain ischemia.mp. OR intracranial hemorrhage.mp.

#3 'Upper Extremity'/exp OR arm.mp. OR hand.mp. OR motor function.mp. OR Fugl-Meyer.mp. OR FMA.mp.

#4 'randomized controlled trial'/exp OR RCT.mp. OR randomized.mp.

#5 #1 AND #2 AND #3 AND #4

**Search terms for Web of Science**

TS=(Mirror Therapy OR mirror visual feedback OR mirror training OR mirror treatment) AND TS=(Stroke OR cerebrovascular accident OR hemiplegia OR brain ischemia OR intracranial hemorrhage) AND TS=(Upper Extremity OR arm OR hand OR motor function OR Fugl-Meyer OR FMA) AND TS=(Randomized Controlled Trial OR RCT OR randomized)

**Search terms for Cochrane Library**

MeSH Terms: Mirror Therapy, Stroke, Upper Extremity, motor function, Fugl-Meyer Assessment

Key Words: mirror visual feedback, mirror training, mirror treatment, cerebrovascular accident, hemiplegia, FMA, RCT, randomized controlled trial

Combination: (Mirror Therapy OR mirror visual feedback OR mirror training OR mirror treatment) AND (Stroke OR cerebrovascular accident OR hemiplegia) AND (Upper Extremity OR arm OR hand OR motor function OR Fugl-Meyer OR FMA) AND (Randomized Controlled Trial OR RCT)

**Table S2.** Baseline characteristics included in the study.

| Inclusion in the Study | Age (in years) | | Gender: Male/Female | | Stroke type (ischemic/hemorrhagic) | | Lesioned side (left/right) | | course of disease | | MMSE | | MAS | | FMA-UE（/66） | | WMFT-T | | WMFT-FA | |
| --- | --- | --- | --- | --- | --- | --- | --- | --- | --- | --- | --- | --- | --- | --- | --- | --- | --- | --- | --- | --- |
|  | MT | control | MT | control | MT | control | MT | control | MT | control | MT | control | MT | control | MT | control | MT | control | MT | control |
| Alsalem 2025 | 54.97 ± 7.11 | | 26 (68.4) / 12 (31.6) | | 28 (73.7) / 10 (26.3) | | 22 (57.9) / 16 (42.1) | | 20.24 ± 28.88 | | 26.50 ± 2.39 | | 1.18 ± 0.24 | | Average Rank 18.66 | Average Rank 20.34 | Average Rank 20.45 | Average Rank 18.55 | Average Rank 17.82 | Average Rank 21.18 |
| Amasyali 2016 | 58.78 ± 10.12 | ES 51.29 ± 12.25；CG 65.38 ± 9.07 | 5 (55.6) / 4 (44.4) | ES 2 (28.6) / 5 (71.4)；CG 4 (50.0) / 4 (50.0) | 9 (100) / 0 (0) | 15 (100) / 0 (0) | 5 (55.6) / 4 (44.4) | ES 2 (28.6) / 5 (71.4)；CG 7 (87.5) / 1 (12.5) | 4.11 ± 2.14 | ES 5.29 ± 2.49；CG 6.50 ± 1.60 | NA | NA | NA | NA | 36.55 ± 17.80 | ES 41.00 ± 17.95；CG 39.87 ± 17.74 |  |  | NA | NA |
| Antoniotti 2019 | 68.2 ± 14.4 | 69.5 ± 14.1 | 14 (70.0) / 6 (30.0) | 12 (60.0) / 8 (40.0) | NA | NA | 13 (65.0) / 7 (35.0) | 13 (65.0) / 7 (35.0) | 0.77 ± 0.22 | 0.72 ± 0.31 | NA | NA | NA | NA | 28.5 ± 21.8 | 30.9 ± 23.9 | NA | NA | NA | NA |
| Arya 2015 | 48.76 ± 13.58 | 42.12 ± 12.52 | 15 (88.2) / 2 (11.8) | 10 (62.5) / 6 (37.5) | 7 (41.2) / 10 (58.8) | 10 (62.5) / 6 (37.5) | 4 (23.5) / 13 (76.5) | 3 (18.8) / 13 (81.3) | 12.88 ± 8.05 | 12.25 ± 5.74 | 25.09 ± 2.80 | 26.01 ± 2.17 | NA | NA | 19.71 ± 7.22 | 18.25 ± 5.43 | NA | NA | NA | NA |
| Chan 2018 | 65.3 ± 11.8 | 64.1 ± 13.3 | 10 (66.7) / 5 (33.3) | 12 (60.0) / 8 (40.0) |  | 10 (66.7) / 5 (33.3) | 10 (66.7) / 5 (33.3) | 12 (60.0) / 8 (40.0) | 0.46 ± 0.22 | 0.42 ± 0.22 | 27.9 ± 2.8 | 25.6 ± 3.1 | NA | NA | 19.2 ± 16.0 | 21.7 ± 15.1 | 92.2 ± 37.8 | 77.6 ± 39.2 | 1.4 ± 0.6 | 1.8 ± 0.7 |
| de Sire 2025 | 71.13 | CIMT 73.05； convention 72.85 | 19 (47.5) / 21 (52.5) | CIMT 21 (52.5) / 19 (47.5)； convention 23 (57.5) / 17 (42.5) | NA | NA | 25 (62.5) / 15 (37.5) | CIMT 25 (62.5) / 15 (37.5)；convention 14 (35.0) / 26 (65.0) | NA | NA | NA | NA | NA | NA | NA | NA | NA | NA | NA | NA |
| Gurbuz 2016 | 60.9 ± 10.9 | 60.8 ± 20.0 | 10 (62.5) / 6 (37.5) | 7 (46.7) / 8 (53.3) | 15 (93.8) / 1 (6.3) | 10 (66.7) / 5 (33.3) | 8 (50.0) / 8 (50.0) | 9 (60.0) / 6 (40.0) | 1.51 ± 1.42 | 1.39 ± 1.24 | NA | NA | NA | NA | 12.8 ± 7.8 | 12.4 ± 8.9 | NA | NA | NA | NA |
| Hsieh 2025 | 49.84 ± 7.70 | DAOT 47.74 ± 9.29； conventionCR 54.19 ± 17.65 | 5 (71.4) / 2 (28.6) | DAOT 0 (0) / 6 (100)； conventionCR 6 (100) / 0 (0) | 5 (71.4) / 2 (28.6) | DAOT 2 (33.3) / 4 (66.7)； conventionCR 3 (50.0) / 3 (50.0) | 5 (71.4) / 2 (28.6) | DAOT 3 (50.0) / 3 (50.0)；CR 3 (50.0) / 3 (50.0) | 14.29 ± 4.61 | DAOT 23.50 ± 18.81； conventionCR 30.17 ± 21.79 | 26.29 ± 2.43 | DAOT 27.83 ± 1.33； conventionCR 27.83 ± 2.93 | NA | NA | 39.14 ± 11.57 | DAOT 37.00 ± 12.18； conventionCR 37.83 ± 15.82 | NA | NA | NA | NA |
| Hsu 2022 | 56.7 ± 11.5 | VR‑MT 52.9 ± 11.8；COT 56.9 ± 13.0 | 7 (41.2) / 10 (58.8) | VR‑MT 8 (44.4) / 10 (55.6)；COT 5 (29.4) / 12 (70.6) | NA | NA | NA | NA | 39.8 ± 28.8 | VR‑MT 30.7 ± 21.1；COT 38.1 ± 26.6 | NA | NA | NA | NA | 32.1 ± 15.2 | VR‑MT 42.3 ± 14.3；COT 34.5 ± 17.4 | NA | NA | NA | NA |
| Kim 2023 | 61.00 ± 5.64 | MTVADG 59.58 ± 5.65；CG 58.75 ± 3.44 | 8 (66.7) / 4 (33.3) | MTVADG 7 (58.3) / 5 (41.7)；CG 7 (58.3) / 5 (41.7) | 7 (58.3) / 5 (41.7) | MTVADG 8 (66.7) / 4 (33.3)；CG 9 (75.0) / 3 (25.0) | 6 (50.0) / 6 (50.0) | MTVADG 7 (58.3) / 5 (41.7)；CG 8 (66.7) / 4 (33.3) | 6.75 ± 2.63 | MTVADG 7.00 ± 2.45；CG 6.67 ± 2.35 | 26.33 ± 2.23 | MTVADG 26.17 ± 2.17 | NA | NA | 41 | MTVADG 43.4；CG 43.2 | NA | NA | NA | NA |
| Lee 2012 | 58.8 ± 12.1 | 55.4 ± 12.2 | 8 (61.5) / 5 (38.5) | 7 (53.8) / 6 (46.2) | NA | NA | 5 (38.5) / 8 (61.5) | 6 (46.2) / 7 (53.8) | 3.5 ± 1.5 | 3.6 ± 1.3 | 25.2 ± 1.5 | 25.6 ± 1.5 | NA | NA | 19.1 | 20.3 | NA | NA | NA | NA |
| Madhoun 2020 | 49.33 ± 10.43 | 53.93 ± 8.76 | 11 (73.3) / 4 (26.7) | 9 (60.0) / 6 (40.0) | 5 (33.3) / 10 (66.7) | 4 (26.7) / 11 (73.3) | 9 (60.0) / 6 (40.0) | 10 (66.7) / 5 (33.3) | 4.13 ± 1.84 | 3.60 ± 1.76 | NA | NA | NA | NA | 19.33 ± 7.62 | 20.60 ± 12.07 | NA | NA | NA | NA |
| Michielsen 2011 | 55.3 ± 12.0 | 58.7 ± 13.5 | 7 (35.0) / 13 (65.0) | 13 (65.0) / 7 (35.0) | 14 (70.0) / 6 (30.0) | 14 (70.0) / 6 (30.0) | NA | NA | 4.7 ± 3.6 | 4.5 ± 2.6 | NA | NA | NA | NA | 39.7 ± 14.1 | 36.4 ± 14.7 | NA | NA | NA | NA |
| Mirela 2015 | 58.2 ± 7.2 | 56.8 ± 8.3 | 4 (57.1) / 3 (42.9) | 4 (50.0) / 4 (50.0) | 54.3 ± 7.9 | 52.2 ± 12.7 | 2 (28.6) / 5 (71.4) | 3 (37.5) / 5 (62.5) | 54.3 ± 7.9 | 52.2 ± 12.7 | NA | NA | NA | NA | 34.1 ± 8.4 | 38.6 ± 6.2 | NA | NA | NA | NA |
| Radajewska 2013 | NA | NA | 18 (60.0) / 12 (40.0) |  | 30 (100) / 0 (0) | 30 (100) / 0 (0) | 15 (50.0) / 15 (50.0) | 15 (50.0) / 15 (50.0) | 10 | 9 | NA | NA | NA | NA | NA | NA | NA | NA | NA | NA |
| Samuelkamaleshkumar 2014 | 48.4 ± 15.58 | 53.9 ± 11.57 | 8 (80.0) / 2 (20.0) | 8 (80.0) / 2 (20.0) | 8 (80.0) / 2 (20.0) | 6 (60.0) / 4 (40.0) | 4 (40.0) / 6 (60.0) | 6 (60.0) / 4 (40.0) | 3.7 ± 1.1 | 4.4 ± 1.4 | 28.2 ± 1.99 | 27.0 ± 2.00 | 0.8 ± 1.8 | 0.8 ± 0.9 | 9.7 ± 10.0 | 4.3 ± 9.9 | NA | NA | NA | NA |
| Wen 2022 | 53.76 ± 11.76 | 57.89 ± 10.74 | 18 (72.0) / 7 (28.0) | 23 (85.2) / 4 (14.8) | 15 (60.0) / 10 (40.0) | 21 (77.8) / 6 (22.2) | 11 (44.0) / 14 (56.0) | 12 (44.4) / 15 (55.6) | 31.00 (16.50‑50.50) | 30.00 (21.00‑60.00) | NA | NA | NA | NA | 19.48 ± 16.62 | 20.59 ± 18.65 | NA | NA | NA | NA |
| Wu 2013 | 54.77 ± 11.66 | 53.59 ± 10.21 | 11 (68.8) / 5 (31.3) | 12 (70.6) / 5 (29.4) | 10 (62.5) / 6 (37.5) | 10 (58.8) / 7 (41.2) |  | 8 (50.0) / 8 (50.0) | 19.31 ± 12.57 | 21.88 ± 15.55 | 29.00 ± 1.00 | 28.06 ± 1.98 | NA | NA | 45.94 ± 8.91 | 44.41 ± 10.69 | NA | NA | NA | NA |
| Yavuzer 2008 | 63.2 ± 9.2 | 63.3 ± 9.5 | 8 (47.1) / 9 (52.9) | 9 (47.4) / 10 (52.6) | 14 (82.4) / 3 (17.6) | 15 (78.9) / 4 (21.1) | 10 (58.8) / 7 (41.2) | 11 (57.9) / 8 (42.1) | 5.4 ± 2.9 | 5.5 ± 2.5 | NA | NA | 1.4 ± 0.5 | 1.7 ± 0.6 | NA | NA | NA | NA | NA | NA |
| Zhang 2021 | 57.0 ± 10.4 | 59.9 ± 11.8 | 15 (50.0) / 15 (50.0) | 18 (60.0) / 12 (40.0) | NA | NA | NA | NA | 0.97 ± 0.26 | 1.03 ± 0.29 | NA | NA | NA | NA | 16.8 ± 5.0 | 15.6 ± 3.9 | NA | NA | NA | NA |
| Zhang 2024 | 58.58 ± 12.71 | CCFES 60.36 ± 10.67；MT+CCFES 62.17 ± 11.88；Control 54.46 ± 10.19 | 3 (25.0) / 9 (75.0) | CCFES 8 (57.1) / 6 (42.9)；MT+CCFES 6 (50.0) / 6 (50.0)；Control 3 (23.1) / 10 (76.9) |  |  | NA | NA | 0.45 ± 0.13 | CCFES 0.67 ± 0.49；MT+CCFES 0.74 ± 0.77；Control 1.10 ± 0.85 | 24.67 ± 2.90 | CCFES 25.21 ± 2.67；MT+CCFES 24.25 ± 3.72；Control 26.46 ± 2.15 | NA | NA | 14.83 ± 13.47 | CCFES 12.86 ± 12.19；MT+CCFES 15.17 ± 13.15；Control 16.69 ± 14.87 | NA | NA | NA | NA |

**Note:** MT, Mirror Therapy (or Motor Training, depending on the original study). ES, Experimental group. CG, Control group. CIMT, Constraint‑Induced Movement Therapy. DAOT, Dynamic Arm Orthosis Training (or a specific active training protocol). CR, Conventional Rehabilitation. VR, Virtual Reality. COT, Conventional Occupational Therapy (or Control group in some studies). MTVADG, a combined intervention (refer to original study for full definition). CCFES, Contralaterally Controlled Functional Electrical Stimulation. MMSE, Mini‑Mental State Examination. MAS, Modified Ashworth Scale. FMA‑UE, Fugl‑Meyer Assessment for Upper Extremity (total score out of 66). WMFT‑T, Wolf Motor Function Test – Time. WMFT‑FA, Wolf Motor Function Test – Functional Ability. Other column headers are self‑explanatory.

**Figure S1.** Literature screening flowchart.

**Figure S2.** Risk of bias results for the included RCTs.

**Figure S3.** Funnel plot for publication bias.
